# Polymers in Technologies of Additive and Inkjet Printing of Dosage Formulations

**DOI:** 10.3390/polym14132543

**Published:** 2022-06-22

**Authors:** Evgenia V. Blynskaya, Sergey V. Tishkov, Konstantin V. Alekseev, Alexandre A. Vetcher, Anna I. Marakhova, Dovlet T. Rejepov

**Affiliations:** 1 V. V. Zakusov Research Institute of Pharmacology, 8 Baltiyskaya St., 125315 Moscow, Russia; evaureus@gmail.com (E.V.B.); sergey-tishkov@ya.ru (S.V.T.); convieck@yandex.ru (K.V.A.); 2 Institute of Biochemical Technology and Nanotechnology, Peoples’ Friendship University of Russia (RUDN), 6 Miklukho-Maklaya St., 117198 Moscow, Russia; agentcat85@mail.ru (A.I.M.); redzhepov-d@rudn.ru (D.T.R.); 3 Complementary and Integrative Health Clinic of Dr. Shishonin, 5 Yasnogorskaya St., 117588 Moscow, Russia

**Keywords:** polymers, additive production, dosage formulations, inkjet printing, 2D printing, 3D printing, 4D printing, smart polymers

## Abstract

Technologies for obtaining dosage formulations (DF) for personalized therapy are currently being developed in the field of inkjet (2D) and 3D printing, which allows for the creation of DF using various methods, depending on the properties of pharmaceutical substances and the desired therapeutic effect. By combining these types of printing with smart polymers and special technological approaches, so-called 4D printed dosage formulations are obtained. This article discusses the main technological aspects and used excipients of a polymeric nature for obtaining 2D, 3D, 4D printed dosage formulations. Based on the literature data, the most widely used polymers, their properties, and application features are determined, and the technological characteristics of inkjet and additive 3D printing are shown. Conclusions are drawn about the key areas of development and the difficulties that arise in the search and implementation in the production of new materials and technologies for obtaining those dosage formulations.

## 1. Introduction

Currently, the development of pharmaceutical technology is taking place in several directions, one of which is the production of personalized dosage formulations (DF). This direction is associated with the need for individual pharmacotherapy for patients taking medicinal products (MP) with a “narrow” therapeutic index, as well as pharmaceutical substances (PS), with accurate data on the concentration of drugs in the blood of various groups of patients and a directly proportional relationship “dose-effect” [1]. In particular, such medicinal products (MP) include cytostatics, aminoglycoside antibiotics and anticonvulsants [2,3,4]. In connection with the development of the presented direction, there is a need to create universal technological methods for the creation of medicinal products (MP) for individual dosing that correspond to the characteristics of metabolism, age, and genetics of the patient. The most promising aspects of this direction are the technologies of additive manufacturing; two-dimensional (2D, inkjet) [5,6,7,8] and three-dimensional (3D) [9,10,11,12,13] printing of MP [14,15]. The versatility and accuracy of placement of liquids with a pharmaceutical substance (PS) are also noted, depending on the application, the relative ease with which the process can be controlled (using the simplest software), and the repeatability of the distribution of liquid volumes. However, 3D printing (additive printing) allows for the production of individual drugs for patients in a wide range of dosages, shapes, and sizes, so it is the main technology for creating personalized dosage formulations.

Nowadays, the addition of adaptive polymers (i.e., smart polymers (SP)), which change their morphology in a predetermined manner in response to the influence of certain factors, provides new options to the previously developed techniques. This concept is called four-dimensional (4D) printing, where time is used as the fourth dimension, since the DF configuration is transformed during a measured time interval. Changes in the DF structure are the result of the use of smart (intelligent) polymers that react to stimuli such as light, temperature, water, or pH.

All of the presented types of technologies are characterized by the employment of a large number of polymeric nature excipients (Es). Each of the Es has a specific functional application, structure, and modifications. Frequently used polymers for traditional dosage formulations find their particular role and are often modified accordingly. Examples of such Es are polylactic acid (PLA), hydroxypropyl methylcellulose (HPMC), Eudragit^®^ (copolymers derived from esters of acrylic and methacrylic acids), polyvinyl alcohol (PVA), chitosan, gelatin, polyvinylpyrrolidone (PVP, Kollidon^®^), etc., which are used to create 3D printed MPs. Therefore, the optimization and selection of polymers are required to manage the processing properties and control frequently appearing problems, such as defects and heterogeneity of properties and PS release [16]. The goal of our review is to consider the properties of polymers used to obtain MP by 2D, 3D, and 4D printing, functional features for each technology, development prospects, and the direction of the search for new materials and their combinations.

## 2. The Methods for Manufacturing Personalized Dosage Formulations

### 2.1. Obtaining DF with 3D Printing

**Figure 1:**
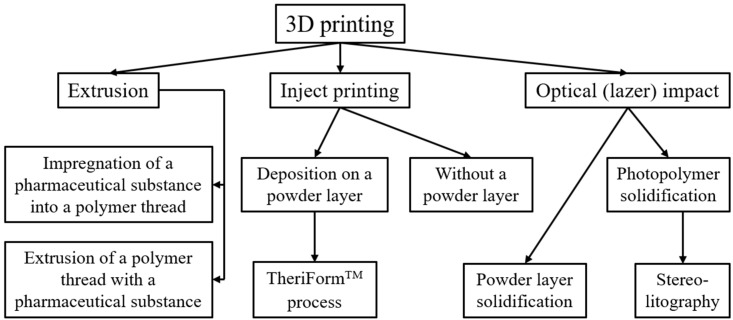
Classification of 3D printing methods.

Three-dimensional printing is a form of additive production in which an object is created by successive layer-by-layer deposition or bonding of materials [17,18]. The advantages of additive methods’ application for the development and production of MP include the ability to gain precise control on the spatial distribution of the PS in the DF, create diverse and complex shapes of the MP, control the dosage of the minimum amount of MP, and reduce the amount of waste. A large number of technologies have been developed for the industrial production of 3D structures, but there are several main methods used for pharmaceutical 3D printing. The technologies used can be classified (Figure 1) based on the occurrence and course of the following main physical processes: extrusion (filament melting), drip (using binder solutions), and laser systems (sintering/solidification) [19].

Each method differs in the structure of the printed object, and the use of 3D printing material. In addition, it defines process characteristics that may be preferred by different PS. The advantages and disadvantages associated with each of the existing approaches can be demonstrated by comparing dimensional accuracy, mechanical properties, surface roughness, assembly speed, and material costs across multiple 3D printing platforms [20].

Some of the main 3D printing technologies worth highlighting are exhibited in Figure 1 and include extrusion printing (fused deposition modeling—FDM), fusion in the powder layer or powder bed fusion (PBF), inkjet printing, and stereolithography (SLA), pressure-assisted microsyringes (PAM) technology, etc. [21]. During the FDM method, the printed layers are obtained from a continuously fed thread of polymer (thermoplastic), which is heated to a molten state for extrusion through a nozzle [22]. After that, the deposited layer cools and solidifies on the platform or top of the previously deposited layer, forming the necessary structure [23,24,25,26]. The PAM method is distinguished by the use of a syringe-extruder for the layer-by-layer application of a viscous material (polymers, hydrogels and aerogels) using a pneumatic piston under pressure [27]. The PBF method can be explained as fusing thin layers of powder material with a laser beam. There is selective laser melting (SLM) and selective laser sintering (SLS), which differ in the degree of energy impact [28]. Inkjet systems for 3D printing, as well as for 2D printing, include continuous inkjet (CIJ) and drop-on-demand printing (DOD), which are described in more detail below [29]. SLA uses exposure to ultraviolet light (or electron beam) on a layer of radiation-sensitive resin or monomer solution in which a free radical chain reaction is initiated to cause polymerization [30].

### 2.2. Preparation of Dosage Formulations Using 2D Inkjet Printing

**Figure 2:**
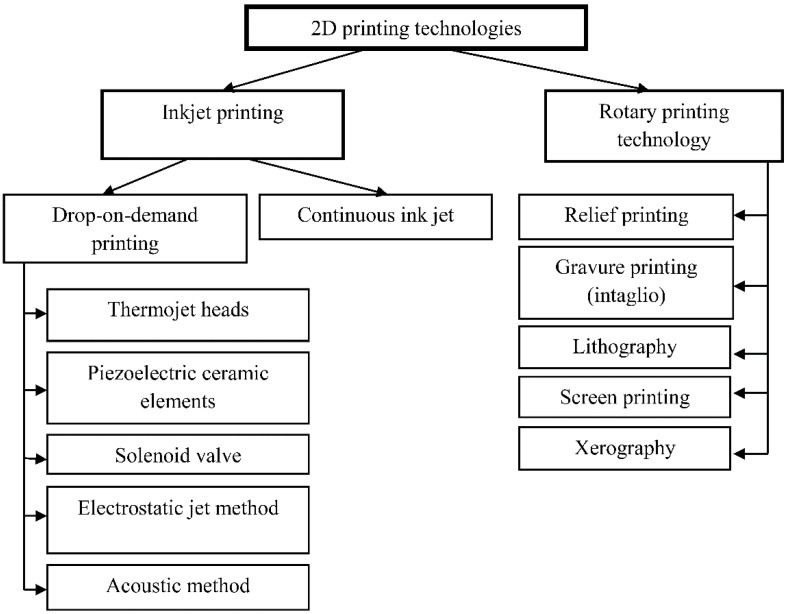
The technologies employed in 2D printing.

The suitable printing technologies can be selected based on the final product requirements and PS properties. Two-dimensional printing technologies are usually divided into inkjet and rotary printing technologies (Figure 2), which differ depending on the specifics of DF production.

Inkjet printing is a general term that covers a wide range of approaches to the formation and placement of small liquid droplets using digital control. Inkjet technology is generally classified as continuous inkjet (CIJ) and on-demand printing (drop-on-demand, DOD) as already mentioned for 3D printing technologies. The types of printing also differ in the physical process by which drops are applied to the printed material. According to [31], CIJ printing consists of passing a continuous flow of liquid through a hole (nozzle), which converts it under the action of surface tension forces into a stream of drops. To obtain a printed pattern, only individual drops from a continuous stream of solutions must be directed to a specific location on the substrate. This is usually achieved by applying an electrical charge to some of the droplets, which then deviate from the main axis of the flow, while passing through an electrostatic field. Some of the drops fall into a special chute and the liquid recirculates in the system.

During the DOD procedure, the liquid is pushed out of the printhead only when externally actuated in response to a signal from the device. The DOD printhead typically contains one or more nozzles in which droplets are ejected by converting kinetic energy from the sources located in the printhead near the nozzle. Many printhead designs use the deformation of a piezoelectric ceramic element for this purpose, while in thermal jet heads, the pressure pulse that ejects the drop is generated by the expansion of a small vapor bubble formed by the action of a small electric heating element in the liquid iTechnologie [32]. There are advantages and disadvantages to both types of device actuation. Piezoelectric printheads can handle a wider range of liquids than thermal printheads (limited to liquids that must vaporize satisfactorily), while the latter can be easier and cheaper to manufacture. Small volumes of liquid can be used for DOD printing, unlike CIJ printing, which requires a significant amount of recirculation, and thus this technique is used in most pharmaceutical research inkjet printers [33,34]. Two-dimensional pharmaceutical printing systems are mainly based on these methods; however, for pharmaceutical applications, other DOD methods, such as solenoid valve and electrohydrodynamic inkjet printing, are also being studied. In solenoid valve inkjet printing, droplet formation is controlled by a valve containing a ferromagnetic piston that opens or closes the flow of solutions between the print head chamber and the nozzle, while generating a magnetic field with an electric current. In addition, there are roto-printing methods, which include contact printing, which requires the physical transfer of a printed pattern onto substrates. Typical rotary techniques, including relief, gravure, lithography (offset), and xerographic printing, have many industrial applications due to their high productivity. However, the lower accuracy of these methods and the complexity of a personalized approach are one of the limiting factors for pharmaceutical applications [31,35].

### 2.3. The Main Ways of 4-D Modification of 3D and 2D Printing

Four-dimensional (4D) printing is defined as the printing of three-dimensional and two-dimensional objects, with the ability to change shape or function under the influence of external stimuli over time [36]. The essential difference between 4D printing and 3D printing is the addition of intelligent design or adaptive materials, which results in a time-dependent deformation of the object. To achieve the presented goal, the printed material must independently transform in form or function when exposed to an external stimulus, such as osmotic pressure, temperature changes, electromagnetic, ultraviolet radiation, etc. [37]. However, the inclusion of additional material adaptation functions in the DF creates additional difficulties in the development process, since 4D printed structures must be preprogrammed based on the transformation mechanism of controlled SPs that include the required deformations of Es. Since most materials for 3D and 2D printing are designed only for the production of rigid, static objects, the choice of Es for 4D printing is a particularly difficult task.

## 3. Polymers for Additive, Inkjet, and 4D Printing

Polymers are the basis for the production of 3D and 2D printed dosage formulations, since they can change the pharmaceutical and technological parameters of finished dosage formulations and intermediates (printing inks, filaments, substrates), and modify the release rate and stability of the PS. For application in this field, polymers of natural and synthetic origin are used. Among natural polymers, gelatin, collagen, alginate, and chitosan are used; however, they sometimes require the presence of cross-linkers to prepare materials for use, which can be cytotoxic [38,39,40,41,42,43]. For those reasons, and due to more suitable processing conditions, synthetic resins are becoming more advantageous for 3D and 2D printing. Table 1 and Table 2 provide a comparative overview of the different printing methods for personalized DF and the polymers used for the MP, respectively.

Polyvinyl alcohol (PVA), polyurethane (PU), polylactide (PLA) and poly(lactide-co-glycolide) (PLGA), hydroxypropyl methicellulose (HPMC), hydroxyethyl cellulose (HEC), polyvinylpyrrolidone (PVP), methacrylate-based polymers, polyethylene glycols, ethylene vinyl acetate, and others are primarily used as the main polymers for extrusion 3D printing.

Polyvinyl alcohol is a common thermoplastic water-soluble polymer and is used for 3D printing, due to its easy water solubility and satisfactory mechanical performance. This polymer is used to prepare DF using commercially available filaments by impregnating PS before printing, after which tablets are created layer by layer [81]. Polyurethane can also be used for FDM 3D printing, in particular sustained-release tablets. Tablets loaded with theophylline and metformin were obtained by this method and showed sustained release [88]. Polylactide is a hydrophobic biodegradable polymer and is, therefore, used in research to produce vaginal rings and PLGA patches.

Using polycaprolactam, intrauterine devices and 3D tablets were prepared, where the scaffold was printed using this polymer and filled with a polyvinyl alcohol-polyacrylic acid hydrogel containing controlled-release indomethacin sodium. In addition, the main use of polycaprolactam and its derivatives, such as polyhydroxyethyl glycosade-co-caprolactam, is in the manufacture of 3D scaffolds for bone regeneration.

Hydroxypropyl methylcellulose is one of the best polymers for making filaments used for FDM, since there are already several examples of the use of various grades of pharmaceutical-grade HPMC for the production of filaments and the subsequent manufacture of tablets by extrusion 3D printing [77,87,89]. In addition, mixtures of HPMC with Kollidon VA 64 (vinylpyrrolidone-vinyl acetate copolymer) were used to produce tablets by stereolithography, where the food pigment Candurin^®^ (Golden Radiance) was used to increase radiation absorption.

For the copolymers obtained from esters of acrylic and methacrylic acids for the preparation of 3D printed DF, water-soluble Eudragit L, S, FS, and E brands were used, which have pH-dependent swelling and PS release. In addition, Eudragit RL and RS, NE polymers can also be used for the controlled release of MP and Eudragit EPO for immediate release. When using these Es, the FDM method produced tablets, implants, and nanocapsules. PVP is another polymer with optimal physical and chemical properties for practical applications in FDM 3D printing, both alone and in mixtures, for example, with methacrylic acid copolymers. PVP was most widely used as a core filler, and Eudragit L100-55 in the shell [90].

Polyethylene glycol diacrylate (PEGDA) can be used to produce hydrogels by 3D printing and in combinations for 4D printing, due to the solubility in water and the photosensitivity of some groups. PEGDA was used as a cross-linking agent to obtain a pH-sensitive hydrogel, and together with PEG as a plasticizer to obtain stereolithographic printing with hydrogelmet [44,91].

For the technology of 2D printing, polymers are used mainly to create substrates on which solutions with PS and Es are applied, so-called “ink”. Water-based solutions are preferred due to their non-toxic nature and suitability for thermal inkjet and piezoelectric printing. In aqueous solutions, the concentration of water-soluble PS can be easily changed to adjust the amount of printed MP. However, many PS have certain solubility limitations. Unlike thermal inkjet printing, piezoelectric printing applies to solutions with non-aqueous solvents, such as ethanol or dimethyl sulfoxide. However, the use of organic solvents should be limited as it requires the removal of residual solvents after printing. In addition, solvents with a low evaporation temperature can clog the nozzle and affect print quality. Therefore, the concentration of the solutions is highly dependent on the solvent used and/or the addition of solubilizing Es or co-solvents. To modify the viscosity, glycerin and Es of a polymeric nature are used (propylene glycol, polyethylene glycols, and hydroxypropyl cellulose), adding them to various pharmaceutical formulations. Other components of the solutions include coloring and taste masking Es, the final composition is determined based on the properties of the PS and the requirements of the printing system.

Substrates are determined as a portable carrier on which the MP solution is printed. Research has often focused on the practical and technical aspects of 2D printing specific formulations, with less attention to the substrate. However, the development of suitable substrates is an important task, since the nature of the substrate can determine the polymorphic shape of any crystals formed upon solvent evaporation. It was not, for example, that the substrate affected the crystallization of naproxen when printing on various solid amorphous dispersions [92].

Table 2 demonstrates the list of suitable different substrates, including edible substrates, such as sugar sheets, polymer and starch films, and non-edible substrates, such as paper and acetate films. The use of ready-made food and pharmaceutical substrates, as well as the development and manufacture of new types of substrates, is becoming an urgent task that should be solved, along with the introduction of 2D printing technology. For pharmaceutical purposes, various substrates often require desired quality characteristics, such as release modification, adsorption, etc. [93].

For 2D printing technologies, sheets of edible paper (rice, corn, wheat) or specially made substrates are usually used as substrates by casting, extrusion from polymeric materials (gelatin, HPMC, etc.) or electro interlacing of polymeric filaments of the material [91].

There are currently a limited number of stimulus-sensitive Es available for 4D printing, as not all adaptive materials can be 3D printed. There are two main types of smart polymer materials that are used in 4D printing, including hydrogels, which swell when exposed to water or other solutes, and shape memory SP. SP responds to a range of stimuli, including temperature, pH, or ultraviolet light.

Hydrogels containing magnetic particles, or ferrogels, are materials sensitive to a magnetic field. An example is an alginate-based scaffold that controls the movement of water from internal pores under the action of a magnetic field, thereby causing the release of cells or PS [94]. Currently, hybrid systems are also being developed that contain several layers of SP that respond to different stimuli; such an approach, for example, was implemented when creating a three-dimensional printed DF that responds to a magnetic field, as well as to changes in pH. This construction was made from a two-layer structure of polyethylene glycol acrylate (PEGDA) and 2-hydroxyethyl methacrylate (PHEMA) hydrogel and contains particles of iron oxide (Fe_3_O_4_), which can move under the action of an external magnetic field to their destination and release the encapsulated MP when pH changes.

Photosensitive materials can change their shape based on photoisomerization and photodegradation in the polymer chain. Similar mechanisms are used in microcapsules with a ring structure, where stereolithographic 3D printing has been used to make injection molds for rings and strips from photosensitive PEGDA resin. Another example of photosensitivity is the use of cross-linked PHEMA functionalized with azobenzene groups, where exposure to light changes the degree of swelling [95]. The use of moisture-sensitive materials generates bending caused by PEG, azobenzene derivatives conjugated to PEG, and agarose films (PCAD AG) [20] or cellulose-based materials [96]. The obtained microcapsules, printed as two-layer structures using 1-[4-(2-hydroxyethoxy)phenyl]-2-hydroxy-2-methyl-1-propan-1-on as a photoinitiator, open under the influence of differences in the swelling characteristics of the layers’ hydrogel and transform their shape to form microstructured objects. A similar principle is adopted by adding a non-swellable but flexible material as a second layer to form connections between rigid linear structures [67].

## 4. Directions of Issue Development

Three-dimensional and two-dimensional technologies for the preparation of DF are a new area for personalization of MP, where polymers play a major role. The main difficulties, especially for 3D and 4D printing, are associated either with the unsatisfactory technological characteristics of the resulting dosage formulations or with the applicability of polymers, due to non-compliance with the regulatory safety requirements for pharmaceutical use. In addition, there are technical difficulties in the use of some polymeric materials, for example, for FDM, due to the high melting point of the polymer, this method applies to a very limited number of PS. Therefore, there is a need to develop polymeric materials with a low melting point, while maintaining the strength of technological characteristics, or to use special technological approaches, such as the encapsulation of PS in nanocapsules [97]. At the same time, the choice of polymeric materials for pharmaceutical FDM printing is very limited. For 2D technology, there is a need to develop substrate compositions with specific adsorption properties, while maintaining the strength and flexibility of the substrate sheets. This effect is achieved by technological methods (electrospinning and lyophilization) or through the use of composite polymeric materials with special characteristics.

## 5. Conclusions

Inkjet, additive printing, and 4D printing technologies are complementary and interdependent technologies, as they perform similar functions with differences in manufacturing complexity, production speed, accuracy, and uniformity. A particularly relevant area for further application is the combination of inkjet printing with SPs to create modified dosage formulations, while taking advantage of 2D printing in terms of low cost and ease of production. Polymeric materials in 2D printing are mainly used to provide adhesive and pharmaceutical-technological characteristics of substrates, as well as to modify the viscosity, surface tension, and other rheological characteristics of inks. Further research should pay special attention to polymers such as PVA, PVP, PEG of various molecular weights, since they have a special effect on both viscosity and surface tension and can modify the strength of the substrates, spreadability on the substrate, drying time, and PS release. In addition, using these materials makes it possible to create DF for modified release, for example, delayed-release mucoadhesive films. A large number of polymeric materials, such as PVA, PLGA, PLA, PVP, HPMC, etc., have been studied for 3D and 4D printing. Often, mixtures of these polymers are used, or with the addition of plasticizers to prevent crystallization, which is especially important for obtaining solid disperse systems with insoluble or poorly soluble substances. In addition, these polymeric materials are especially promising when creating floating delivery systems using 3D printing. The main directions for the application of those Es are the provision of mechanical and strength characteristics under the FDA, in combination with the modification of MP release, since a feature of 3D printing is the ability to achieve pre-calculated release kinetics by changing the spatial configuration of the distribution of various materials. The effect shown most clearly demonstrates the use of various types of methacrylic acid copolymers in the shell and core of the tablet or combination with other materials. The admission into the composition of SP will make it possible to achieve targeted delivery and use of MP for many more complex cases, with the need to adjust therapy compared to traditional dosage formulations.

The key features for the use of new types of materials and technologies for inkjet and 3D printing, including SP, are the solution to many regulatory and technological problems in providing comprehensively personalized patient therapy. At the same time, there are already approaches from relatively simple inkjet printing methods to 3D printing with polymers with modified properties.

## Figures and Tables

**Table 1:** Comparative overview of different 2D and 3D printing methods.

Technology	Source Material	Polymer(s) Exploited	Mode and Resolution	References
		**3D**		
Fused deposition modeling FDM	Filament	Thermoplastic polymers, such as polycarbonate, ABS, PLA and nylon	Extrusion and deposition, 50–200 (Rapide Lite 500)	[23,24,25]
Stereolithography SLA	Liquid photo polymer	Photopolymer (epoxy or acrylate based resin)	Laser scanning and UV induced curing, 10 (DWSLAB XFAB)	[44]
Selective laser sintering SLS	Powder	Polykaprolaktam, polyamides, etc.	Laser scanning and heat induced sintering, 80 (Spo230 HS)	[45,46]
Inkjetprinting	Powder	Any powder Es, as well as polymers that correct the rheological characteristics of the liquid	Drop-on-demand binder printing, 100–250 (Plan B, Ytec3D)	[47]
Pressure assisted microsyringes (PAM)	Liquid polymer	Polymer with effective viscosity to form a suspension, with optimum shear and compression yield strength to avoid nozzle blockage, e.g., HPMC, carbomers, etc.	The piston of the pouring machine creates a pressure of ~3–5 bar and squeezes out the polymer; (3D printer (Fab@Home) resolution 25 µm)	[27]
3D printing by drop deposition (drop-on-drop)	Liquid polymer	Polymer system. PS must be soluble in a volatile solvent, using “ink” with an optimum viscosity between injector throughput and liquid leakage (PEG, HPMC, PLHA).	Drop-on-demand binder printing, 100–250 (Plan B, Ytec3D)	[48,49]
		**2D**		
Piezoelectric printing	Substrate	Substrate material: HPMC. Ink material: 40:60 (*v*/*v*) PEG 400 and ethanol; water; 5% (*w*/*v*) PEG 8000 in water	25 µm	[50,51,52]
Thermal inkjet printing	Substrate	Substrate material: sodium PVA-CMC, HPMC. Ink material: 10:90 (*v*/*v*) glycerol and water; 30:70 (*v*/*v*) PPG and water; 10:20:70 (*v*/*v*/*v*) glycerol, methanol and water; DecoColour^®^ yellow (Uchida of America Corp., Torrance, CA, USA) food solutions; 10% (*v*/*v*) food red solutions in 10:90 (*v*/*v*) mixture of glycerol and water	9–10 µm	[33,53,54,55]
Drop deposition using a pump	Substrate	Substrate material: HPMC. Ink material: ethanol, FS/PVP complex	Wide range adjustable	[56,57]
Electrodynamic printing	Substrate	Substrate material: HPMC. Ink material: PEG 400; 2% (*w*/*v*) sodium lauryl sulfate in PEG 400	15–70 µm	[58]
Flexography	Substrate	Substrate material: HPMC. Ink material: PEG 400; 5:95 (*w*/*w*) HPC in ethanol; 5:95 (*w*/*w*) HPC in water	30–75 µm	[50,59]

**Table 2:** Polymers used to obtain dosage formulations for personalized use.

Polymer	Drug Delivery System	Printing Technology	References
Hydroxypropylmethylcellulose (HPMC)	Matrix tablets	3D printing; by extrusion printing	[60]
	Orally dispersible film	2D printing (substrate material)	[33,52,57,58,59]
Poly(lactic-co-glycolic acid) (PLGA)	Microsphere, capsules, tablets, nanospheres	3D printing; by extrusion printing	[61,62,63,64]
Copolymers of methacrylic acid (Eudragit^®^ RLPO, Eudragit^®^ RL, Eudragit^®^ E100)	Tablets (“rapid retard” systems, separable tablets, enteric dual pulsatile release, dual pulsatile release)	3D printing, dropping powder: TheriForm™ process	[47,49]
PLGA (poly(lacto-co-glycolic acid)) and PLA (poly-L-lactide), PEG/HPMC	Matrix tablet	3D printing by drop deposition (drop-on-drop)	[34,65]
HPMC, Methocel^®^ K100M/Carbopol^®^ 974P NF.	Matrix tablet	3D printing (pressure-assisted microsyringes, PAM)	[27]
Polyvinyl alcohol, Eudragit^®^ RL, RS	Matrix tablet, controlled release system	3D printing (fused-deposition modeling, FDM)	[66]
pNIPAM-AAc	Nanoparticles of poly(*N*-isopropylacrylamide-coacrylic acid) (pNIPAM-AAc), polypropylene fumarate (PPF), iron oxide (Fe_2_O_5_)	4D printing	[67]
Methacrylated polycaprolactone	Poly (e-caprolactone) (PCL) dimethyl acrylate, 2,4,6-trimethylbenzoyl-diphenylphosphine oxide (TPO) as a photoinitiator, vitamin E to prevent premature crosslinking, yellow 3GP toner	Stereolithography (Freeformpico 2 SLA digital laser printer)	[68]
PVA/PEG hydrogel	Polyvinyl alcohol (PVA)-polyethylene glycol (PEG) double sided hydrogel	4D printing	[69]
Acrylic acid copolymers	Epoxidized soybean oil acrylate contains three major fatty acid residues (stearic, oleic and linoleic acids) with pendant alkane groups that can freeze and improve shape hold at −18 °C.	Stereolithography (modified Solidoodle^®^ 3D printer platform)	[70]
PEGDA/PHEMA	PEG-acrylate (PEGDA), iron(II, III) oxide (Fe_5_O_4_); 2-hydroxyethyl methacrylate (PHEMA) layer, micro and nanoparticles	4D printing	[71]
Vinyl caprolactam/PE hydrogel	Vinyl caprolactam, polyethylene, epoxy diacrylate oligomer, Irgacure^®^ 819	StratasysConnex 500 multipurpose 3D printer	[72]
Polyethylene glycol based systems (PEG 400:ethanol, PEG 8000:water	Orally dispersible film	2D piezoelectric printing (ink)	[50,52]
Polyethylene glycol 400		2D electrodynamic printing, flexography (ink)	[33,58]
Poly (methacrylates) (Eudragit)	Nanocpasules, tablets	3D printing; extrusion printing method; stereolithographic printing	[73]
Poly (ethylene glycol) diacrylate (PEGDA)	Hydrogel	3D; 4D printing; stereolithography	[74]
Polyvinyl alcohol (PVA)	Tablets, capsule	3D printing; by extrusion printing	[75]
Polylactic acid (PLA)	Nanofibres	3D printing; by extrusion printing	[76]
Polyvinylpyrrolidone (PVP or Kollidon^®^)	Tablets; orally dispersible tablets (ODT)	3D printing; by extrusion printing; 3D printing, dropping on TheriFlash™ powder	[77,78,79]
Poly (ε-caprolactone) (PCL)	Tablets, carbon nanotubes	3D printing; extrusion printing method; laser sintering method	[80]
Polyurethane (PU)	Tablets, hydrogel	3D printing; extrusion printing method; 3D printing (pressure-assisted microsyringes, PAM; (inner diameter, 260 µm, and outer diameter, 463.6 µm)	[81,82]
Pluronic	Hydrogel	3D bioprinting with UV crosslinking	[83]
Poly(*N*-(2-hydroxypropyl) methacrylamide-mono/dilactate)-polyethylene glycol triblock copolymer (M15P10)	Thermosensitive hydrogel	3D bioprinting; by extrusion printing	[84]
Ethylcellulose (EC)	Tablets	3D printing; hot melt extrusion	[85]
Ethylene vinyl acetate	T-shaped intrauterine systems (IUS) and sub-cutaneous rods (SR)	3D printing; by extrusion printing	[86]
Poly(methylmethacrylate) (PMMA)	Tablets	3D printing by drip deposition	[78]
Methacrylic/cellulosic polymers	Tablets	3D printing; hot melt extrusion	[34,87]

## Data Availability

No new data were created or analyzed in this study. Data sharing is not applicable to this article.
